# Editorial: Recent Advances in the Immunology of Helminth Infection – Protection, Pathogenesis and Panaceas

**DOI:** 10.3389/fimmu.2021.663753

**Published:** 2021-03-17

**Authors:** Kara J. Filbey, Constance A. M. Finney, Paul R. Giacomin, Mark C. Siracusa

**Affiliations:** ^1^ Lydia Becker Institute of Immunology and Inflammation, Faculty of Biology, Medicine and Health, The University of Manchester, Manchester, United Kingdom; ^2^ Department of Biological Sciences, University of Calgary, Calgary, AB, Canada; ^3^ Australian Institute of Tropical Health and Medicine, James Cook University, Cairns, QLD, Australia; ^4^ Department of Medicine, Rutgers New Jersey Medical School, Rutgers University, Newark, NJ, United States

**Keywords:** helminth, immuno-modulation, immunity, immunomodulators, pathogen

## Introduction

Helminths (parasitic worms) are a diverse group of organisms that utilize a wide range of species as their intermediate and definitive hosts. The nematodes consist of the whipworms, roundworms, hookworms and filarial worms, and these sit alongside the platyhelminth flatworms (or blood flukes) and tapeworms - all of which have species that cause serious disease in humans. Some species have free living stages, others rely on insect vectors for transmission, while some can reproduce to release live larval stages within their mammalian host. The diversity of infection route, larval migration within the host and the location of the adult parasite have major implications for the pathology and immune responses elicited by each species.

Here, we briefly outline the contributions to the Research Topic Recent Advances in the Immunology of Helminth Infection – Protection, Pathogenesis and Panaceas.

## Protection

Many millions of people are infected with helminths world-wide (1), and there is, as yet, no effective vaccine against any helminth species. Indeed, the protective immune responses needed to kill and clear worms from the host are still to be fully elucidated. Zawawi and Else outline the challenges involved in helminth vaccine development, and review recent findings on the identification of suitable vaccine candidates as well as the results of recent pre-clinical and clinical trials in the context of soil-transmitted helminths. Silvane et al. investigate the protective immune responses induced by vaccination of mice with a peptide from a well-characterized protease inhibitor molecule made by the liver fluke *Fasciola hepatica.* Using a novel adjuvant formulation, FhKTM/CpG-ODN/Coa-ASC16 induced enhanced type 1 and type 17 responses and reduced liver pathology after *F. hepatica* infection.

The lung and intestinal tract are the two tissues most often associated with helminth larval migration and adult dwelling respectively. They are both key sites of immune-mediated protection against helminth parasites and are inextricably linked *via* mucosal immune cross-talk (2). In this Research Topic, Weatherhead et al. review the critical interface of pulmonary immune responses and helminth infections, and Faniyi et al. outline the roles of chemosensory epithelial cells within the gastrointestinal tract in host protection against helminths. Illustrating further the complex nature of the intestinal immune system, original research by Mayer et al. shows that discrete areas of the intestine mount differing Th1 and Th2 responses to helminth antigens. The authors were able to demonstrate that antigens from *Schistosoma mansoni* eggs and *Heligmosomoides polygyrus* are transported from the gut to particular mesenteric lymph nodes by separate subsets of dendritic cells, inducing distinct responses, suggesting a highly antigen-dependent and site-specific response to helminth infections.

Type 2 innate lymphoid cells (ILC2s) have been of particular interest to helminth immunologists in recent years (3), and the review by Miller and Reinhardt focusses in detail on the inflammatory subset of ILC2s that arise in the lungs after infection with the model hookworm *Nippostrongylus brasiliensis*.

Of course, much of what we know regarding host immune responses to helminth infections has been gained from laboratory infection models. In their extensive review, Colombo and Grencis outline the models traditionally used to investigate soil-transmitted helminth infections in laboratory animals and highlight the major differences between these and natural infections of humans and wild rodents. They go on to discuss the potential benefits of using the ‘trickle-infection’ method for modelling more natural infections in the laboratory.

## Helminth Immunomodulators

An area of great interest in the field has been the immunomodulatory effects of helminths on their hosts, and their potential use as therapeutic agents in inflammatory diseases (4). Recent technological advances have hastened the discovery of individual molecules secreted by helminths, giving a deeper insight into just how helminth-driven immunomodulation may be occurring. Three broad-ranging reviews by Bobardt et al., Wiedemann and Voehringer and Ryan et al. take an in-depth look at the molecules made by a range of helminth species to impact the immune response against them, thus allowing them to successfully invade and persist in their hosts for long periods of time.

Two original research papers examined individual helminth-derived immunomodulatory molecules in precise detail. Firstly, Chauche et al. explain mechanistically how a truncated form of Alarmin Release Inhibitor from *H. polygyrus* (HpARI) binds to the cytokine interleukin (IL)-33 to prevent its degradation and enhance its actions *in vivo.* In contrast to the full-length form of HpARI which binds IL-33 and suppresses its actions, the truncated form HpARI_CCP1/2 amplifies IL-33-dependent responses to *N. brasiliensis* infection and fungal allergen administration. In a study by Jin et al. a protein from the excretory-secretory (ES) products of *Trichinella spiralis* - thioredoxin peroxidase-2 (TsTPX2) – is found to potently activate macrophages to a protective M2 phenotype, with the adoptive transfer of these antigen-activated macrophages limiting adult worm numbers significantly.

Most of the helminth-derived immunomodulatory molecules characterized to-date are proteins. However, recently the potential importance of micro(mi)RNAs released in extracellular vesicles secreted by helminths has been investigated (5) (and see below). In this vein, Ricafrente et al. use an innovative data-mining approach to identify potential gene targets for *F. hepatica* miRNAs within innate immune cells, thus providing information for the development of novel strategies for controlling liver fluke infection.

## Panaceas and Pathogenesis

Moving from the identification of individual molecules to modulating inflammation and immunopathology in *in vivo* models of disease, Cleenewerk et al. outline the immunomodulatory effects of live infection with *S. mansoni* alongside its ES products, and discuss the potential for use in the clinic. Research by Yang et al. utilizes miRNA-containing extracellular vesicles from *T. spiralis* to reduce inflammatory immune responses and alleviate intestinal epithelial barrier damage in a model of colitis.

Two papers used the well-characterized gastrointestinal helminth *H. polygyrus* to ameliorate inflammation in diverse models of disease in mice. White et al. report that the potent skewing towards a type 2 immune response during helminth infection was protective in experimental autoimmune encephalitis (EAE). Importantly, this protection was ablated in mice lacking the interleukin-4/13 receptor alpha chain (IL-4Rα) and protection was not linked with regulatory T cell activity. Additionally, Filbey et al. show that infection significantly reduces skin inflammation in a model of contact hypersensitivity, likely *via* suppression of innate cell recruitment to the skin after allergen sensitization. Neutrophil-attracting chemokine levels, and neutrophil numbers in the skin of mice treated with the contact sensitizer dibutyl phthalate fluorescein isothiocynate (DBP-FITC), were significantly decreased by helminth infection.

Three reviews in this Research Topic expertly outline the impact of helminth infection on a diverse range of human health concerns. Firstly, Rajamanickam et al. focused on parameters associated with non-diabetic obesity and assessed outcomes in a human cohort before and 6 months after anthelminthic treatment. The predominant helminth species endemic to the Tamil Nadu region of southern India is the soil-transmitted GI helminth *Strongyloides stercoralis* which appeared to protect people from the inflammatory immune signature of non-diabetic obesity, a phenomenon that was reversed after worm clearance. An important review by Chetty et al. looks at the often overlooked field of female reproductive health, focusing on the impact of helminth infection on sexually-transmitted infections, female reproductive tract pathology and reproduction. Finally, Fonte et al. take a timely look at the possible impacts of concomitant infection with helminths and SARS-CoV-2.

## Final Words and Acknowledgments

An estimated quarter of the world’s population is infected with helminths, which clearly have wide-ranging and potent effects on the immune systems of their hosts. Much is yet to be discovered regarding the responses elicited by and the exciting potential uses of helminths. As guest Topic Editors we hope that the Research Topic Recent Advances in the Immunology of Helminth Infection – Protection, Pathogenesis and Panaceas has offered an up-to-date insight into the diverse and collaborative field of helminth immunology. We would like to thank all of the contributing authors, editors, and especially all of the reviewers who dedicated their precious time in what has proved to be a most challenging year.

## Author Contributions

All authors contributed equally to this manuscript. All authors contributed to the article and approved the submitted version.

## Funding

KF is funded by The Kennedy Trust for Rheumatology Research (via the Senior Research Fellowship of Dr John Grainger). CF is funded by the Canadian Foundation for Innovation (CFI - JELF 33617) and the Natural Sciences & Engineering Research Council of Canada (NSERC - RGPIN/04305-2014). PG is funded by the Australian National Health and Medical Research Council (NHMRC - 1105300). MS is funded by the National Institutes of Health (NIH - R01 AI131634, R01 AI123224 and R01 AI151599-01).

## Conflict of Interest

PG is a co-founder of Paragen Bio, a biotech company that aims to translate and commercialise hookworm-derived proteins for inflammatory diseases. This has previously involved investment from pharma (Abbvie Inc) and venture capitalist firms (OneVentures, Brandon Capital).

MS is founder and president of the biotech company NemaGen Discoveries.

The remaining authors declare that the research was conducted in the absence of any commercial or financial relationships that could be construed as a potential conflict of interest.
